# Hypercapnia and lung function parameters in chronic obstructive pulmonary disease

**DOI:** 10.1186/s12890-024-03151-1

**Published:** 2024-07-16

**Authors:** Lukas Gernhold, Claus Neurohr, Konstantinos Tsitouras, Nina Lutz, Selina Briese, Alessandro Ghiani

**Affiliations:** 1 Department of Pulmonology and Respiratory Medicine, Lung Center Stuttgart – Schillerhoehe Lung Clinic GmbH, affiliated with Robert-Bosch-Hospital GmbH (Bosch Health Campus), Auerbachstr. 110, Stuttgart, 70376 Germany; 2 Comprehensive Pneumology Center (CPC-M), Member of the German Center for Lung Research (DZL), Munich, Germany

**Keywords:** COPD, Hypercapnia, Pulmonary function tests, Lung function parameters

## Abstract

**Background:**

In advanced chronic obstructive pulmonary disease (COPD), hypercapnia may occur due to severe bronchial obstruction with lung hyperinflation. Non-invasive ventilation (NIV) provides the standard of care intended to achieve physiological PCO_2_ levels, thereby reducing overall mortality. The present study aimed to evaluate pulmonary function parameters derived from spirometry (forced vital capacity [FVC], forced expiratory volume in 1 s [FEV1]), body plethysmography (residual volume [RV], total lung capacity [TLC]), and lung diffusion capacity for carbon monoxide (single-breath method [DCO-SB], alveolar-volume corrected values [DCO-VA]) as predictors of chronic hypercapnia in patients with advanced COPD.

**Methods:**

This monocentric, retrospective observational study included 423 COPD patients. Receiver operating characteristic (ROC) curve analysis and cross-validation were used to assess lung function parameters’ diagnostic accuracy for predicting chronic hypercapnia, with the resulting performance expressed as area under the ROC curve (AUROC). We performed univariable and multivariable binary logistic regression analysis to determine if these parameters were independently associated with chronic hypercapnia, with probabilities reported as odds ratios [OR] with 95% confidence intervals [95%CI].

**Results:**

FVC% (AUROC 0.77 [95%CI 0.72–0.81], *P* < 0.01) and FEV1% (AURIC 0.75 [95%CI 0.70–0.79], *P* < 0.01) exhibited reasonable accuracy in the prediction of chronic hypercapnia, whereas lung diffusion capacity performed poorly (AUROC 0.64 [95%CI 0.58–0.71] for DCO-SB%, *P* < 0.01). FVC% (OR 0.95 [95%CI 0.93–0.97], *P* < 0.01) and FEV1% (OR 0.97 [95%CI 0.94–0.99], *P* = 0.029) were the only parameters associated independently with chronic hypercapnia in logistic regression analysis. FVC and FEV1 thresholds that best separated hypercapnic from normocapnic subjects reached 56% and 33% of predicted values.

**Conclusions:**

Routinely collected pulmonary function parameters, particularly FVC% and FEV1%, may predict chronic hypercapnia during COPD progression.

**Supplementary Information:**

The online version contains supplementary material available at 10.1186/s12890-024-03151-1.

## Background

Chronic obstructive pulmonary disease (COPD) is common in industrialized nations [1]. Within the next decade, COPD deaths are expected to increase further and become the third leading cause of death worldwide by 2030 [2]. A majority of these patients perceive their quality of life to be inferior to that of patients with cardiovascular disease or diabetes. The advanced stages of the disease are characterized by symptom burdens similar to those associated with malignancies [3, 4].

The progression of COPD may result in chronic hypercapnic respiratory failure caused by deteriorating respiratory mechanics associated with an increase in airway obstruction and progressive lung hyperinflation [5]. These patients benefit from non-invasive ventilation (NIV), significantly reducing mortality and improving quality of life [6, 7]. It is recommended that NIV be started in stable COPD patients with chronic hypercapnia at a partial pressure of carbon dioxide (PCO_2_) level greater than 52 millimeters of mercury (mmHg), with the objective of reducing PCO_2_ to physiological values [6]. However, frequently, an acute exacerbation of COPD (ECOPD) results in acute hypercapnia and respiratory acidosis [8], for which NIV is highly effective in avoiding the need for intubation with invasive mechanical ventilation [9]. For these patients, NIV should only be used long-term if hypercapnia persists for more than two weeks following the exacerbation [7].

COPD patients with chronic hypercapnia who do not exhibit acute symptoms usually start NIV later in their disease course. According to a data evaluation of COPD patients with GOLD disease stages III and IV, a significant number of subjects present with untreated chronic hypercapnia [10], a condition associated with an increased risk of mortality [11]. Identifying such patients as early as possible will allow prompt NIV introduction, probably improving their survival. However, in most cases, chronic hypercapnia is diagnosed by chance when patients present with clinical symptoms such as exertional dyspnea.

There is currently scarce information about the factors that predict chronic hypercapnia development in COPD [12, 13]. Sufficient early risk stratification for chronic hypercapnia lacks valid parameters that would permit a transition into justified, closer clinical monitoring to start NIV as early as possible. According to prior research, a low forced expiratory volume seems to be associated with chronic hypercapnic respiratory failure in such patients [14].

The present study aimed to evaluate pulmonary function parameters derived from spirometry, body plethysmography, and lung diffusion capacity for carbon monoxide as predictors of chronic hypercapnia in patients with advanced COPD.

## Methods

This monocentric, retrospective, observational cohort study was conducted at the Schillerhoehe Lung Clinic (Robert-Bosch Hospital GmbH, Germany). Project approval was granted by the local institutional review board for human studies (Ethics Committee of the State Chamber of Physicians of Baden-Württemberg, Germany, file number F-2022-136) and was performed according to the Declaration of Helsinki principles. Because data were evaluated retrospectively and pseudonymously, the institutional review board waived informed consent requirements. We adhered to STROBE guidelines for reporting observational studies [15].

### Patient selection

The recording period is five years, from January 2018 to December 2022. We assessed adult patients with a confirmed COPD diagnosis according to the GOLD definition [16] and with a complete set of pulmonary function tests (spirometry, body plethysmography), blood gas analysis, and carbon dioxide lung diffusion capacity (whenever available).

Exclusion criteria were as follows: History of ECOPD within six weeks of the examination (treated with systemic steroids or antibiotics), body mass index (BMI) > 35 kg/m^2^, the introduction of domiciliary NIV before 2018, any other severe lung diseases (e.g., combined emphysema and pulmonary fibrosis, severe pulmonary hypertension with a mean pulmonary artery pressure > 35 mmHg on right heart catheterization), lung cancer, or previous lung resection surgery.

### Data collection

Data was collected from the hospital’s electronic medical records and charting systems (iMedOne, Telekom Healthcare Solutions, Bonn, Germany). We evaluated patients` baseline demographics, clinical characteristics, comorbidities, pulmonary function tests, blood gas analysis, respiratory support such as long-term oxygen therapy (LTOT) or NIV, and current inhaled medications (long-acting ß_2_-agonists, long-acting anticholinergics, and corticosteroids).

### Classification of outcomes

Patients were divided into two groups according to their actual daytime PCO_2_ as determined by capillary or arterial blood gas analysis. Hypercapnia was defined based on the physiological threshold of > 45 mmHg, equal to 6.0 kilopascals (kPa). As the introduction of domiciliary NIV is typically triggered by higher values [6], additional analyses were performed based on the clinically relevant threshold of > 52 mmHg (6.9 kPa).

### Candidate predictors of hypercapnia

We focused our analysis on lung function parameters derived from spirometry (FVC, FEV1), body plethysmography (RV, TLC), and lung diffusion capacity for carbon monoxide (DCO-SB, DCO-VA).

Forced vital capacity (FVC) refers to the lung volume that can be exhaled at maximum speed after maximum inspiration [17]. The FVC is primarily used to measure the loss in lung capacity associated with restrictive lung diseases such as pulmonary fibrosis. The forced expiratory volume (FEV1) refers to the amount of air the subject can forcefully expel within the first second following maximum deep inspiration [17]. FEV1 primarily serves to determine the severity of airflow limitation in obstructive pulmonary diseases such as COPD or asthma. Residual volume (RV) is the volume remaining in the lungs following maximum forceful expiration [17], preventing the alveoli from closing at end-expiration. Total lung capacity (TLC) represents the volume of gas in the lungs after maximal inhalation. FVC and FEV1 are measured via spirometry, whereas RV and TLC require additional body plethysmography.

A subject’s carbon monoxide lung diffusing capacity (single-breath DCO-SB *versus* alveolar volume-corrected DCO-VA, referring to the Krogh index) reflects the amount of gas transported through its alveoli by diffusion in a given time period, which is determined by the size of the alveolar space and the thickness of the alveolar membrane [17, 18]. Numerous factors, such as pulmonary fibrosis, emphysema, pulmonary hypertension, and anemia, can reduce diffusion capacity.

Pulmonary function testing was conducted following the standards of the American Thoracic Society [17, 19].

### Statistical analysis

Descriptive and frequency statistics were employed to compare demographics and clinical characteristics between patients with and without hypercapnia. A Chi-square or Fisher’s exact test was used to compare categorical variables. Depending on the continuous variables` homogeneity of variance, determined by the Kolmogorov-Smirnov normality test, differences between groups were analyzed through Student’s *t*-test or Mann-Whitney *U*-test.

Receiver operating characteristic (ROC) curve analysis and 2-times repeated, 5-fold cross-validation were used to assess the parameters’ diagnostic accuracy and internal validity for predicting chronic hypercapnia and to determine the parameters’ thresholds (the criterion associated with the Youden index) [20] that best separate hypercapnic from non-hypercapnic individuals. The resulting performance of each parameter was expressed as area under the ROC curve (AUROC), sensitivity, specificity, positive/negative predictive value, accuracy, positive/negative likelihood ratio (PLR/NLR), diagnostic odds ratio (DOR), F_1_ score, and Matthews correlation coefficient (MCC) [21]. Correlations between lung function parameters and spontaneous breathing PCO_2_ were determined through Spearmans` coefficient of rank correlation (*ρ*). We performed binary logistic regression analysis to determine if pulmonary function tests were independently associated with chronic hypercapnia and to estimate the probability of hypercapnia. The multivariable model used forward selection and included variables deemed clinically significant a priori (age, gender, and obesity) and those lung function parameters with a *P* value of less than 0.2 in bivariate analysis. Hosmer & Lemeshow and Nagelkerke R^2^ were used to evaluate the model’s goodness of fit. Probabilities are reported as odds ratios (OR) with 95% confidence intervals (95%CI). Finally, we performed linear and multiple regression analysis to formulate equations for estimating spontaneous breathing PCO_2_ based on those lung function parameters found to be independently associated with chronic hypercapnia.

Sensitivity analyses were conducted for selected tests using the clinically relevant PCO_2_ threshold of > 52 mmHg. Since there were no comparable studies on prolonged ventilated lung transplant recipients to determine sample size, we recruited patients to the maximum extent possible. We performed two-tailed tests; statistical significance was indicated by *P* < 0.05. The analyses were conducted with MedCalc^®^ software v20.305 (Ostend, Belgium).

## Results

### Baseline characteristics

**Fig. 1:**
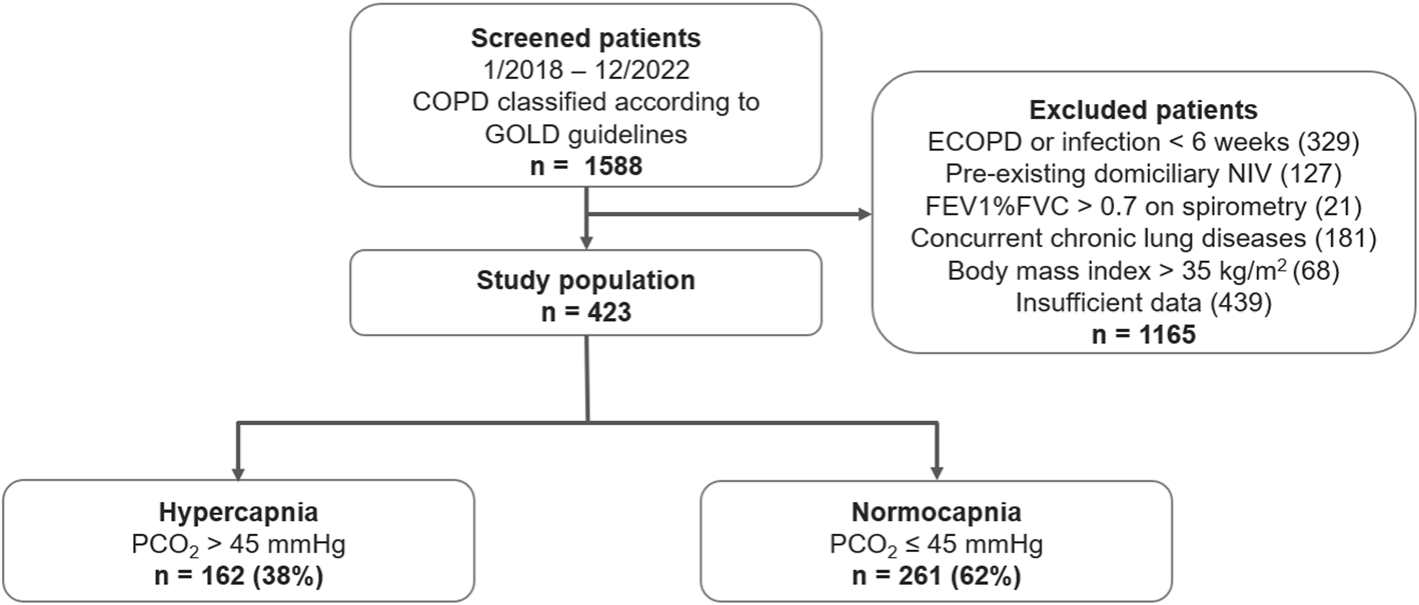
Patient flow diagram. *Abbreviations*: *COPD *chronic obstructive pulmonary disease, *ECOPD *exacerbations of COPD, *NIV *non-invasive ventilation, *FEV1%FVC *Tiffeneau index

The present study screened 1588 patients, of whom 424 were included in the analysis (Fig. 1). The median age of the group was 70 years [IQR 63–77 years]; 165 (39%) of the participants were female, 162 (38%) were hypercapnic (based on the physiological PCO_2_ threshold), and there were no significant differences between the two cohorts in terms of comorbid diseases. Gender-specific distributions were not apparent.

In hypercapnic patients, 93% received LTOT, and 74% received NIV. However, only 47% of normocapnic patients were treated with LTOT. In contrast, 103 of 106 patients (97%) received NIV when PCO_2_ levels were higher than 52 mmHg (the threshold of clinical relevance) (Table 1).

**Table 1:** Clinical characteristics – comparison of patients with and without chronic hypercapnia

Clinical characteristics	All patients (*n* = 423)	Hypercapnia (*n* = 162)	Normocapnia (*n* = 261)	*P* value^a^
Age (years)	70 (63–77)	70 (63–77)	70 (63–77)	0.757^*b*^
Female sex	165 (39.0)	66 (40.7)	99 (37.9)	0.565^*c*^
Body mass index (kg/m^2^)	23.9 (20.6–27.6)	23.7 (20.2–28.1)	23.9 (20.8–27.4)	0.925^*b*^
*Obesity (BMI ≥ 30 kg/m*^*2*^*)*	51 (12.1)	20 (12.3)	31 (11.9)	0.886^*c*^
Smoking history	409 (96.7)	160 (98.8)	249 (95.4)	0.061^*c*^
GOLD stage III-IV	367 (86.8)	157 (96.9)	210 (80.5)	**< 0.01**^***c***^
GOLD stage IV	215 (50.8)	116 (71.6)	99 (37.9)	**< 0.01**^***c***^
Long-term oxygen therapy	275 (65.0)	152 (93.8)	123 (47.1)	**< 0.01**^***c***^
Non-invasive ventilation	121 (28.6)	121 (74.7)	0 (0.0)	**< 0.01**^***c***^
**Inhaled treatments**				
LABA or LAMA	9 (2.1)	3 (1.9)	6 (2.3)	0.757^*c*^
ICS monotherapy	3 (0.7)	0 (0.0)	3 (1.1)	0.171^*c*^
LABA/LAMA	175 (41.4)	78 (48.1)	97 (37.2)	**0.026**^***c***^
LABA/ICS	7 (1.7)	1 (0.6)	6 (2.3)	0.188^*c*^
LABA/LAMA/ICS	207 (48.9)	76 (46.9)	131 (50.2)	0.513^*c*^
No inhaled treatment	22 (5.2)	4 (2.5)	18 (6.9)	**0.047**^***c***^
**Comorbidities**				
Chronic heart failure	68 (16.1)	27 (16.7)	41 (15.7)	0.795^*c*^
Coronary artery disease	100 (23.6)	33 (20.4)	67 (25.7)	0.213^*c*^
Hypertension	215 (50.8)	89 (54.9)	126 (48.3)	0.183^*c*^
Diabetes mellitus	53 (12.5)	26 (16.0)	27 (10.3)	0.085^*c*^
Obstructive sleep apnea	30 (7.1)	13 (8.0)	17 (6.5)	0.557^*c*^

Continuous variables are presented as median (– interquartile range [IQR]), categorical variables are presented as numbers (%)

^a^*P* value for differences between patients with and without chronic hypercapnia

^*b*^Mann-Whitney *U*-test

^*c*^Chi-squared test

*Abbreviations*: *BMI *body mass index, *GOLD *Global Initiative for Chronic Obstructive Lung Disease, *LABA *long-acting β_2_-agonists, *LAMA *long-acting muscarinic antagonists, *ICS *inhaled corticosteroids

### Lung function parameters

There was a significant difference in FVC% (49% [41–58%] *versus* 64% [55–78%], *P* < 0.01) and FEV1% (27% [21–33%] *versus* 37% [28–46%], *P* < 0.01) between hypercapnic and normocapnic individuals. RV differences were less pronounced, and TLC was comparable between groups. Patients with hypercapnia exhibited significantly lower lung diffusion capacity, as well as a lower pH and an increased bicarbonate level in their blood gas analyses (Table 2).

**Table 2:** Pulmonary function parameters and blood gas analysis – comparison of patients with and without chronic hypercapnia

Spirometry	All patients (*n* = 423)	Hypercapnia (*n* = 162)	Normocapnia (*n* = 261)	*P* value^a^
FEV1%FVC (%)	49 (43–56)	48 (42–55)	49 (43–57)	0.251^*b*^
FVC (L)	1.95 (1.50–2.58)	1.65 (1.30–2.00)	2.26 (1.74–2.84)	**< 0.01**^***b***^
FVC%	59 (47–72)	49 (41–58)	64 (55–78)	**< 0.01**^b^
FEV1 (L)	0.85 (0.67–1.14)	0.70 (0.60–0.90)	0.96 (0.77–1.30)	**< 0.01**
FEV1%	32 (26–42)	27 (21–33)	37 (28–46)	**< 0.01**^***b***^
**Body plethysmography**				
RV (L)	4.98 (3.90–6.40)	5.18 (4.10–6.79)	4.90 (3.83–6.10)	**< 0.01**
RV%	213 (172–263)	226 (177–280)	206 (169–254)	**< 0.01**^***b***^
TLC (L)	7.34 (6.04–8.50)	7.11 (5.80–8.50)	7.38 (6.16–8.54)	0.325^*b*^
TLC%	121 (106–136)	122 (105–138)	121 (106–133)	0.687^*b*^
**Lung diffusion capacity**				
DCO-SB (mmol*min^−1^*kPa^−1^)	2.77 (1.89–3.72)	2.23 (1.60–3.28)	2.83 (2.12–3.81)	**0.011**^***b***^
DCO-SB%	32 (24–45)	26 (18–37)	34 (26–47)	**< 0.01**^***b***^
DCO-VA (mmol*min^−1^*kPa^−1^*L^−1^)	0.60 (0.43–0.82)	0.51 (0.38–0.81)	0.61 (0.45–0.82)	0.083^*b*^
DCO-VA%	45 (31–62)	37 (21–59)	48 (34–64)	**< 0.01**^***b***^
**Blood gas analysis**				
PCO_2_ (mmHg)	42 (38–53)	56 (50–63)	39 (36–42)	–
PO_2_ (mmHg)	68 (63–75)	68 (61–78)	68 (63–73)	0.827^*b*^
pH	7.40 (7.38–7.43)	7.38 (7.35–7.40)	7.42 (7.40–7.44)	**< 0.01**^***b***^
HCO_3_ (mmol/L)	27 (25–29)	29 (27–32)	25 (24–27)	**< 0.01** ^***b***^

Continuous variables are presented as median (– interquartile range [IQR]). The parameters of lung diffusion capacity were available in 234 patients (55%), 58 (36%) of whom had hypercapnia

^a^*P* value for differences between patients with and without chronic hypercapnia

^*b*^Mann-Whitney *U*-test

^*b*^Chi-squared test

*Abbreviations*: *FEV1%FVC *Tiffeneau index, *FVC *forced vital capacity, *FEV1 *forced expiratory volume in 1 s, *RV *residual volume, *TLC *total lung capacity, *DCO-SB *single-breath lung diffusion capacity for carbon monoxide, *DCO-VA *transfer coefficient for carbon monoxide (Krogh index)

### Predictors of hypercapnia

**Fig. 2:**
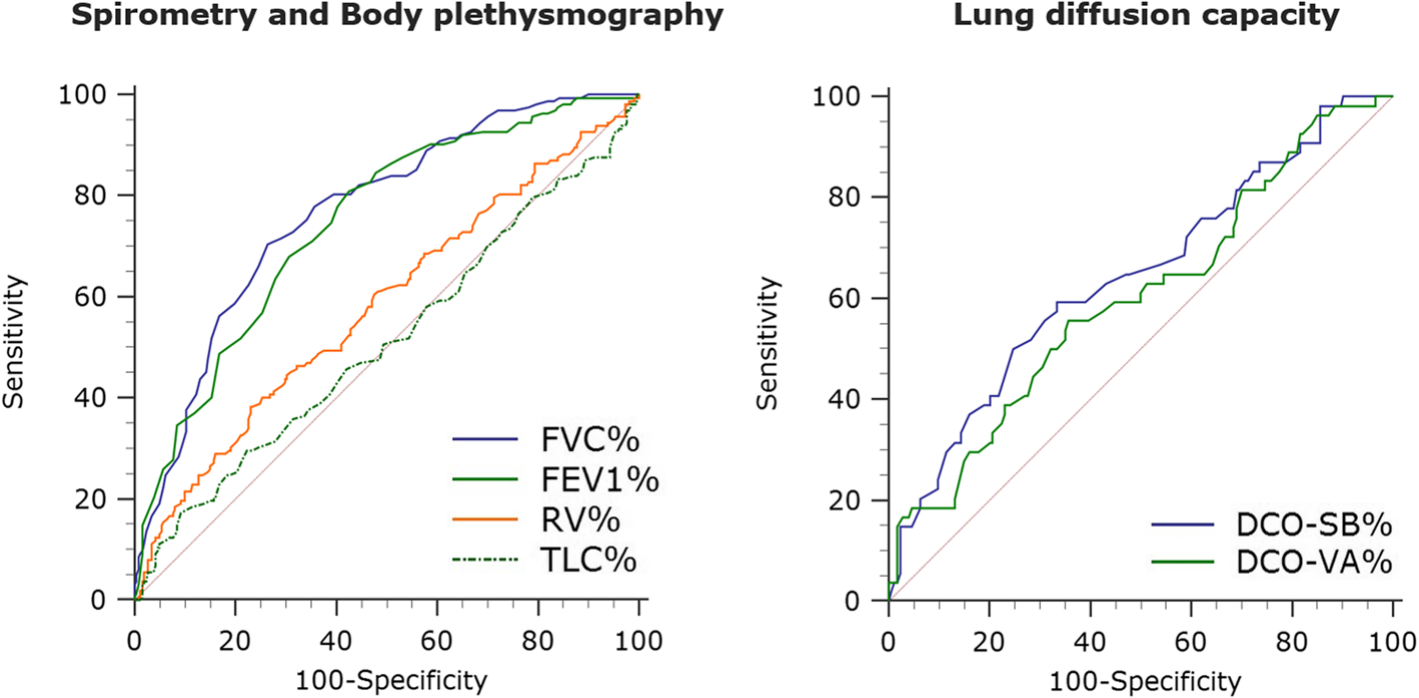
Comparison of ROC curves for selected pulmonary function parameters analyzed to predict chronic hypercapnia. *Abbreviations*: *FVC *forced vital capacity, *FEV1 *forced expiratory volume in 1 s, *RV *residual volume, *TLC *total lung capacity, *DCO-SB *single-breath lung diffusion capacity for carbon monoxide, *DCO-VA *transfer coefficient for carbon monoxide (Krogh index)

ROC curve analysis and cross-validation demonstrated reasonable accuracy in discriminating hypercapnic and normocapnic individuals by FVC% (AUROC 0.77 [95%CI 0.72–0.81], DOR 7.9, MCC 0.41) and FEV1% (AUROC 0.75 [95%CI 0.70–0.79], DOR 6.8, MCC 0.34), while DCO-SB% performed poorly (AUROC 0.64 [95%CI 0.58–0.71], DOR 5.0, MCC 0.17) (Fig. 2; Table 3, Additional file 1: Table S1). Similar results were obtained when sensitivity analyses were conducted based on the 52 mmHg PCO_2_ threshold for defining clinically relevant hypercapnia (Additional file 1: Table S2).

**Table 3:** Cross-validated performance of pulmonary function parameters analyzed to predict chronic hypercapnia

Parameter (threshold)	Sensitivity	Specificity	PPV	NPV	Accuracy	PLR	NLR	DOR	F_1_	MCC
FVC% (56%)	71 (52–85)	71 (57–83)	61 (49–72)	80 (69–87)	71 (61–81)	2.7 (1.6–4.6)	0.4 (0.7 − 0.2)	7.9	0.65	0.41
FEV1% (33%)	75 (57–88)	60 (46–73)	54 (44–64)	79 (67–87)	66 (55–77)	2.0 (1.6–2.8)	0.4 (0.8 − 0.2)	6.8	0.62	0.34
RV% (250%)	38 (22–56)	70 (56–82)	45 (30–61)	65 (57–74)	58 (47–69)	1.4 (0.7–2.8)	0.9 (1.2–0.6)	1.6	0.40	0.09
TLC% (135%)	33 (21–47)	72 (61–79)	48 (27–67)	64 (52–71)	55 (44–66)	1.7 (0.6–5.1)	0.9 (2.0-0.7)	2.0	0.31	0.07
DCO-SB% (27%)	49 (17–81)	71 (52–85)	31 (16–52)	84 (72–90)	66 (50–80)	2.0 (0.8–5.4)	0.7 (1.5 − 0.4)	5.0	0.37	0.17
DCO-VA% (36%)	44 (14–75)	70 (53–84)	26 (12–50)	83 (72–89)	64 (48–77)	1.4 (0.7–4.4)	0.8 (1.6 − 0.5)	2.7	0.30	0.11

Results of 2-times repeated, 5-fold cross-validation. Mean metrics of diagnostic accuracy (with 95% confidence intervals) based on threshold values associated with the *Youden index* (presented as the mean of the thresholds derived from the training sets)

*Abbreviations*: *PPV *positive predictive value, *NPV *negative predictive value, *PLR *positive likelihood ratio, *NLR *negative likelihood ratio, *DOR *diagnostic odds ratio, *F1 *F1 score, *MCC *Matthews` correlation coefficient, *FVC *forced vital capacity *FEV1 *forced expiratory volume in 1 s, *RV *residual volume, *TLC *total lung capacity, *DCO-SB *single-breath lung diffusion capacity for carbon monoxide, *DCO-VA *transfer coefficient for carbon monoxide (Krogh index)

Based on the results of cross-validation, FVC% and FEV1% thresholds that best separated hypercapnic from normocapnic subjects reached 56% and 33% of predicted values (Table 3).

### Rank correlations

**Fig. 3:**
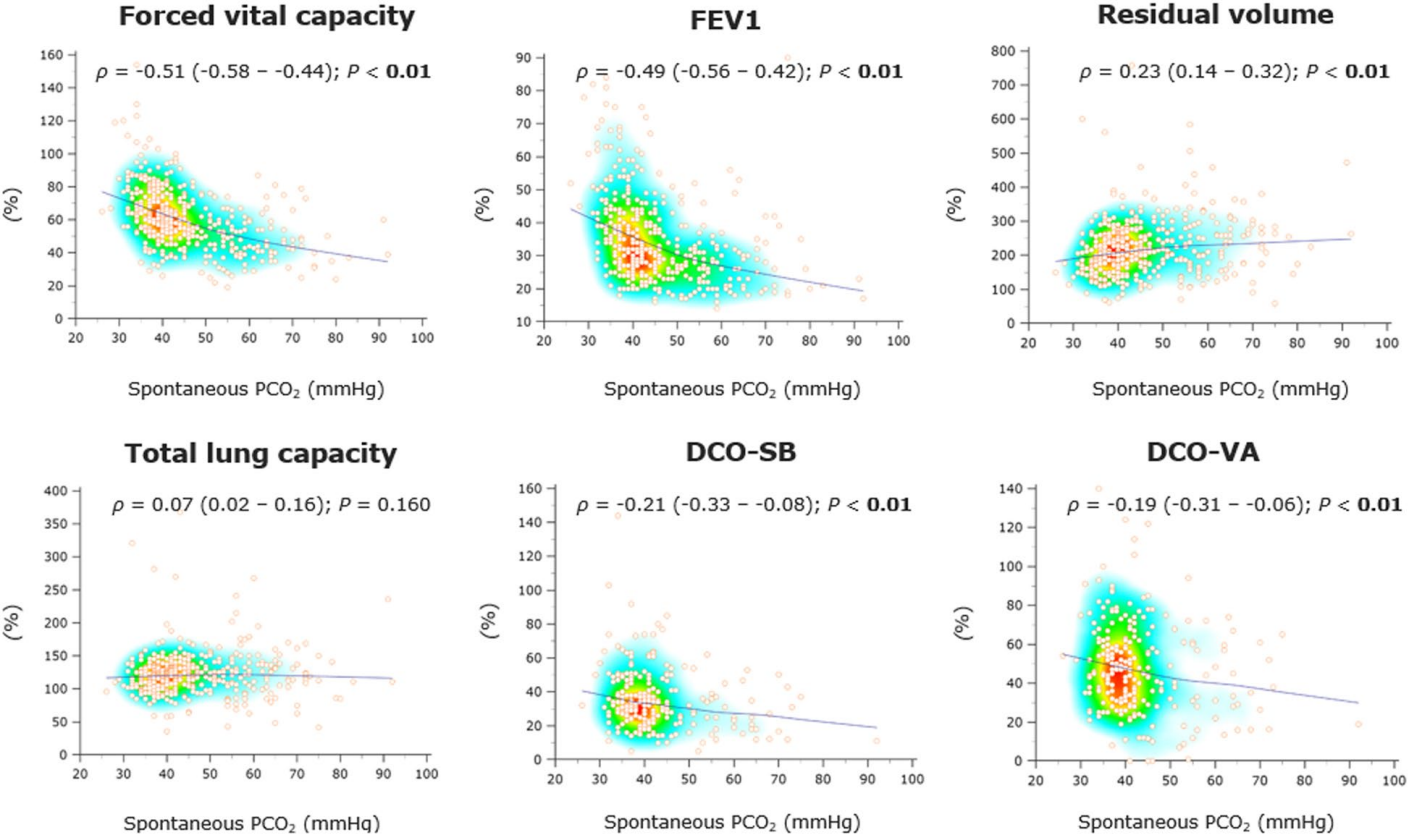
Rank correlations of selected pulmonary function parameters with spontaneous breathing PCO_2_. The heat map of Spearman’s rank correlation coefficients (*ρ*) with the LOESS (Local Regression Smoothing) trendline. *Abbreviations: ρ*, Spearman’s correlation coefficient (with 95% confidence interval); FEV1, forced expiratory volume in 1 s; DCO-SB, single-breath lung diffusion capacity for carbon monoxide; DCO-VA, transfer coefficient for carbon monoxide (Krogh index)

Regarding FVC, FEV1, RV, and DCO-SB, there was a significant correlation between these variables and spontaneous PCO_2_, which was particularly strong for FVC% (Spearmans` *ρ* = -0.51 [95%CI -0.58 – -0.44], *P* < 0.01) and FEV1% (*ρ* = -0.49 [-0.56 – -0.42], *P* < 0.01). In contrast, this was not observed with TLC or DCO-VA (Fig. 3, Additional file 1: Table S3).

### Regression analysis results

**Fig. 4:**
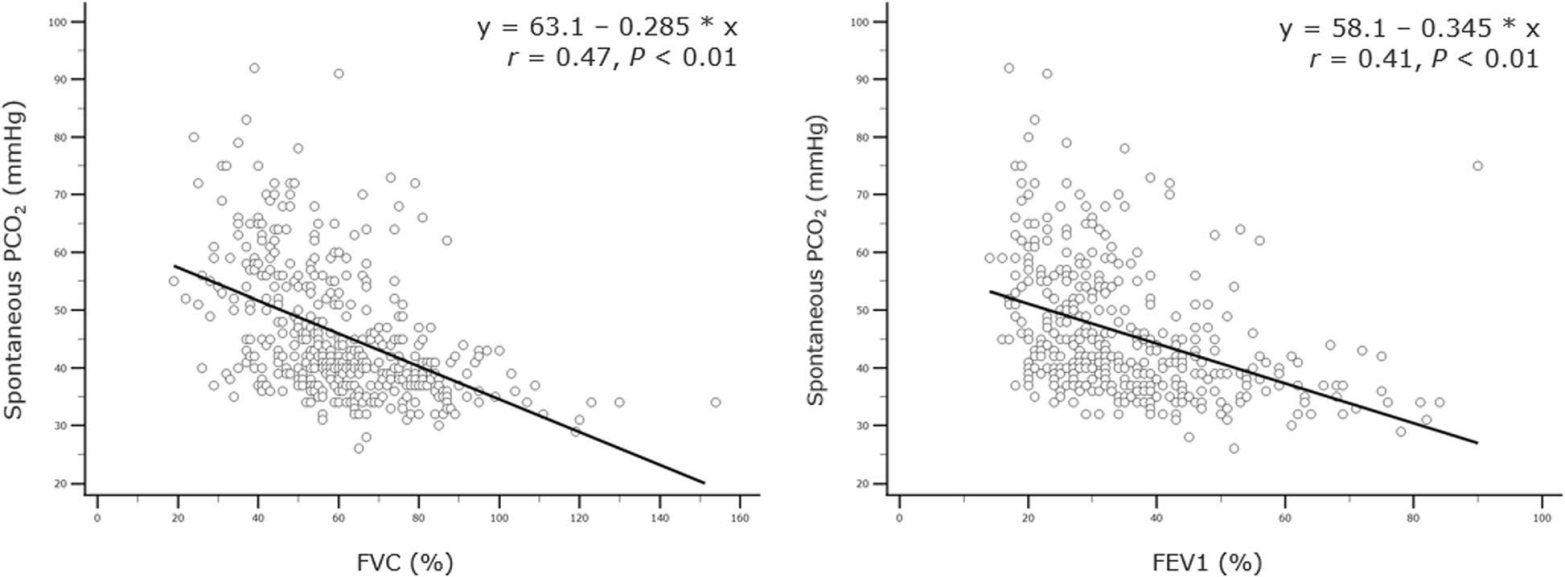
PCO_2_ as a function of FVC% and FEV1%: Linear regression analysis. *Abbreviations*: *FVC *forced vital capacity, *FEV1 *forced expiratory volume in 1 s

In univariable binary logistic regression analysis, FVC%, FEV1%, and RV% were independently related to chronic hypercapnia. After adjusting for age, gender, and presence of obesity (BMI ≥ 30 kg/m^2^), only FVC% (OR 0.95 [95%CI 0.93–0.97], *P* < 0.01) and FEV1% (OR 0.97 [95%CI 0.94–0.99], *P* = 0.029) remained in the final multivariable model (Additional file 1: Table S4-S5). Moreover, both linear and multiple regression analyses showed a significant relationship between FVC% and FEV1% concerning spontaneous PCO_2_ (Fig. 4, Additional file 1: Table S6).

## Discussion

This study aimed to identify pulmonary function tests that predict the likelihood that patients with COPD will develop chronic hypercapnia, which is the primary indicator of whether they should receive domiciliary NIV, a treatment proven to reduce overall mortality. Statistically significant correlations and independent relationships were found between spirometric parameters and actual PCO_2_ levels in patients without a history of acute exacerbations within the previous six weeks. Specifically, FVC% and FEV1% showed reasonable accuracy in identifying patients with and without hypercapnia, as defined by the physiological PCO_2_ threshold of 45 mmHg. Optimal cut-off values for hypercapnia prediction were 56% and 33% of predicted FVC% and FEV1%, respectively. The results of sensitivity analyses were comparable when using 52 mmHg PCO_2_ as the threshold for clinically relevant hypercapnia, which usually results in the introduction of NIV.

It is known that chronic hypercapnia in the context of respiratory insufficiency is associated with an increased mortality rate [22]. The development of hypoxemia or hypercapnia in COPD can be explained by structural changes to the airways, alveoli, and mechanisms of alveolar ventilation [5] in conjunction with a mismatch in ventilation and perfusion [23]. Hypercapnia is characterized by chronically overloaded inspiratory respiratory muscles, referred to as the respiratory pump. With COPD progression, the resistance of the airways increases, resulting in progressively hyperinflated lungs [5]. Due to this obstruction, mechanical stress (pressure) is placed on the respiratory pump. Accordingly, this implies that the lower the FEV1, the higher the airway resistance and, consequently, the respiratory pump’s work of breathing. Upon exhaustion, the alveolar ventilation necessary to maintain normocapnia can no longer be provided. In light of this, it is reasonable that decreasing FEV1 may indicate the development of hypercapnia. Indeed, previous studies have shown that the decrease in FEV1 correlates with the rise in PCO_2_ [14]. However, the present study is the first to evaluate the diagnostic accuracy of pulmonary function tests in predicting chronic hypercapnia, demonstrating that FEV1% and, even more interestingly, FVC% have predictive value for the development of hypercapnia. We defined hypercapnia as PCO_2_ above 45 mmHg (the physiological threshold); however, the introduction of NIV is typically performed when the PCO_2_ exceeds 52 mmHg. Nevertheless, the physiological threshold is a reasonable choice, as many patients with daytime borderline hypercapnia may experience significant increases in PCO_2_ during sleep, which may also result in the initiation of NIV [24].

Currently, the FVC serves primarily as a parameter for assessing restrictive lung diseases, in which a decline indicates the progression of interstitial lung disease [25] and is predictive of patient mortality [26]. However, FVC has not yet been described as a prognostic factor or an indicator of progression in COPD. Based on the present study’s findings, a reduction in ventilated lung volume, as measured by FVC, may also contribute to chronic respiratory failure due to obstructive lung disease. In this scenario, FVC reduction may be more than just an indicator of lung hyperinflation severity, given that RV, another measure of lung hyperinflation, was less accurate at predicting chronic hypercapnic respiratory failure. Theoretically, FVC may be a more accurate indicator of actual functional lung size than RV, with decreasing FVC – indicative of “shrinking” functional lung volume – causing a faster and shallower breathing pattern [12], thereby aggravating dead-space ventilation (which equals reduced ventilatory efficiency) and lung hyperinflation [5]. Evidence suggests that respiratory muscle dysfunction contributes to hypercapnia development [27], primarily due to unfavorable stretching of muscle fibers resulting from lung hyperinflation, adversely affecting their mechanical efficiency [5].

The present findings indicate that lung diffusion capacity is also involved in developing hypercapnia, even though it is not as accurate in diagnosing hypercapnia as FVC1% and FEV%. A decreased diffusion capacity is also associated with reduced ventilatory efficiency, resulting in a higher minute ventilation required to maintain the same PCO_2_ [28, 29]. Consequently, the respiratory pump is under more intense mechanical stress, probably causing exhaustion and chronic respiratory failure.

Several methods have been established for monitoring and following up on COPD patients. The BODE index combines various parameters (body mass index, airflow obstruction, dyspnea, and exercise capacity) and is more predictive of patient mortality than its components alone [30]. However, lung function parameters such as FVC% and FEV1% should be considered more carefully when evaluating chronic hypercapnic respiratory failure. Although spirometry is unlikely to be used as a screening tool [31﻿], it is an essential follow-up test owing to its cost-effectiveness, precision, and objective nature. As per GOLD report 2023, there is no clear recommendation regarding spirometry monitoring and follow-up interval after detecting progressive declines in FEV1% [16]. Generally, one year is considered an appropriate time interval. In light of the potential prognostic value of pulmonary function tests, it may be reasonable to use a shorter interval of six months or even three months for those with a decline in FVC% or FEV1% below the thresholds determined in the present study to prevent missing the onset of clinically relevant hypercapnia. Moreover, there is evidence that FVC and FEV1 are predictive factors for mortality irrespective of smoking status [32, 33], suggesting that regular spirometry should be performed in patients with declining values below critical thresholds.

This study has limitations. Although it involves a large sample size of predominantly advanced COPD GOLD Stage III and IV, it is undoubtedly limited by its retrospective single-center design in that external validation is required to verify the conclusions.

## Conclusions

To summarize, hypercapnia in COPD progression was accurately predicted by spirometric parameters collected during routine care of COPD patients. To detect clinically significant hypercapnia promptly, the control intervals for spirometry should be narrowed in patients with COPD stages 3 and 4 with declining FVC% or FEV1% below critical thresholds.

### Supplementary Information

Supplementary Material 1.

Supplementary Material 2.

## Data Availability

The datasets used and analyzed are available from the corresponding author upon reasonable request.

## Abbreviations

95%CI: 95% confidence interval
AUROC: Area under the receiver operating characteristic curve
BMI: Body mass index
COPD: Chronic obstructive pulmonary disease
DCO-SB: Single-breath CO lung diffusing capacity
DCO-VA: Alveolar volume-corrected CO lung diffusing capacity (Krogh index)
DOR: Diagnostic odds ratio
ECOPD: Exacerbations of COPD
FEV1: Forced expiratory volume
FVC: Forced vital capacity
GOLD: Global Initiative for Chronic Obstructive Lung Disease
IQR: Interquartile range
kPa: Kilopascals
LTOT: Long-term oxygen therapy
MCC: Matthews` correlation coefficient
mmHg: Millimeters of mercury
NIV: Non-invasive ventilation
NLR: Negative likelihood ratio
OR: Odds ratio
PCO_2_: Partial carbon dioxide pressure
PLR: Positive likelihood ratio
ROC curve: Receiver operating characteristic (curve)
RV: Residual volume
TLC: Total lung capacity
