# Supplementary material for: Hypercapnia and lung function parameters in chronic obstructive pulmonary disease
Source: BMC Pulm Med. 2024 Jul 16;24:345. doi: 10.1186/s12890-024-03151-1 (PMC11251095; doi:10.1186/s12890-024-03151-1)
Supplement: Supplementary file 1 — Supplementary Material 1. [file 12890_2024_3151_MOESM1_ESM.pdf]

Hypercapnia and lung function parameters in chronic obstructive pulmonary disease

Lukas Gernhold, MD; Claus Neurohr, MD; Konstantinos Tsitouras, MD; Nina Lutz; Selina Briese; and Alessandro Ghiani, MD

Additional file 1

- **Table S1:** Area under the ROC curve of pulmonary function parameters regarding the prediction of chronic hypercapnia (Page 1)
- **Table S2:** Sensitivity analysis: Area under the ROC curve of pulmonary function parameters regarding the prediction of chronic hypercapnia with a PCO_2 > 52 mmHg (Page 2)
- **Table S3:** Rank correlations of pulmonary function parameters with spontaneous breathing PCO_2 (Page 3)
- **Table S4:** Variables associated with chronic hypercapnia – Results of univariable and multivariable binary logistic regression analysis (all patients; N = 423) (Page 4)
- **Table S5:** Variables associated with chronic hypercapnia – Multivariable binary logistic regression model (all patients; N = 423) (Page 5)
- **Table S6:** Linear and multiple regression models (all patients; N = 423) (Page 6)

Table S1: Area under the ROC curve of pulmonary function parameters regarding the prediction of chronic hypercapnia

**Spirometry**	**AUROC (95%CI)**	***P* value**
FVC	0.74 (0.70 – 0.78)	**< 0.01**
FVC%	0.77 (0.72 – 0.81)	**< 0.01**
FEV1 (L)	0.74 (0.70 – 0.79)	**< 0.01**
FEV1%	0.75 (0.70 – 0.79)	**< 0.01**
**Body plethysmography**		
RV (L)	0.58 (0.53 – 0.62)	**< 0.01**
RV%	0.58 (0.53 – 0.63)	**< 0.01**
TLC (L)	0.53 (0.48 – 0.58)	0.334
TLC%	0.51 (0.46 – 0.56)	0.695
**Lung diffusion capacity**		
DCO-SB ($mmol*min^{-1}*kPa^{-1}$)	0.61 (0.55 – 0.68)	**0.011**
DCO-SB%	0.64 (0.58 – 0.71)	**< 0.01**
DCO-VA ($mmol*min^{-1}*kPa^{-1}*L^{-1}$)	0.58 (0.51 – 0.64)	0.100
DCO-VA%	0.63 (0.57 – 0.69)	**< 0.01**

Legend

The accuracy of each index in predicting chronic hypercapnic respiratory failure presented as the area under the ROC curve with 95% confidence intervals. The parameters of lung diffusion capacity were available in 234 patients (55%), 58 (36%) of whom had hypercapnia.

Abbreviations: AUROC, area under the receiver operating characteristic curve; 95%CI, 95% confidence interval; FVC, forced vital capacity; FEV1, forced expiratory volume in 1 second; RV, residual volume; TLC, total lung capacity; DCO-SB, single-breath lung diffusion capacity for carbon monoxide; DCO-VA, transfer coefficient for carbon monoxide (Krogh index).

Table S2: Sensitivity analysis: Area under the ROC curve of pulmonary function parameters regarding the prediction of chronic hypercapnia with a PCO_2 > 52 mmHg

**Spirometry**	**AUROC (95%CI)**	***P* value**
FVC (L)	0.74 (0.69 – 0.78)	**< 0.01**
FVC%	0.77 (0.73 – 0.81)	**< 0.01**
FEV1 (L)	0.73 (0.68 – 0.77)	**< 0.01**
FEV1%	0.74 (0.70 – 0.78)	**< 0.01**
**Body plethysmography**		
RV (L)	0.56 (0.52 – 0.61)	0.062
RV%	0.57 (0.52 – 0.63)	**0.047**
TLC (L)	0.52 (0.47 – 0.57)	0.530
TLC%	0.51 (0.46 – 0.57)	0.844
**Lung diffusion capacity**		
DCO-SB (mmol*min^{-1}*kPa^{-1})	0.63 (0.57 – 0.69)	**0.014**
DCO-SB%	0.65 (0.58 – 0.71)	**< 0.01**
DCO-VA (mmol*min^{-1}*kPa^{-1}*L^{-1})	0.56 (0.49 – 0.62)	0.306
DCO-VA%	0.61 (0.55 – 0.68)	**0.033**

Legend

The accuracy of each index in predicting chronic hypercapnia with a PCO_2 > 52 mmHg presented as the area under the ROC curve with 95% confidence intervals. The parameters of lung diffusion capacity were available in 234 patients (55%), 58 (36%) of whom had hypercapnia.

Abbreviations: AUROC, area under the receiver operating characteristic curve; 95%CI, 95% confidence interval; FVC, forced vital capacity; FEV1, forced expiratory volume in 1 second; RV, residual volume; TLC, total lung capacity; DCO-SB, single-breath lung diffusion capacity for carbon monoxide; DCO-VA, transfer coefficient for carbon monoxide (Krogh index).

Table S3: Rank correlations of pulmonary function parameters with spontaneous breathing PCO_2

**Spirometry**	**Spearman`s correlation coefficient (*ρ*)**	***P* value**
FVC	- 0.454 (- 0.527 – - 0.375)	**< 0.01**
FVC%	- 0.510 (- 0.578 – - 0.436)	**< 0.01**
FEV1 (L)	- 0.473 (- 0.544 – - 0.396)	**< 0.01**
FEV1%	- 0.493 (- 0.562 – - 0.417)	**< 0.01**
**Body plethysmography**		
RV (L)	0.203 (0.110 – 0.292)	**< 0.01**
RV%	0.228 (0.136 – 0.317)	**< 0.01**
TLC (L)	- 0.002 (- 0.097 – 0.093)	0.968
TLC%	0.069 (- 0.027 – 0.163)	0.160
**Lung diffusion capacity**		
DCO-SB ($mmol*min^{-1}*kPa^{-1}$)	- 0.170 (- 0.293 – - 0.041)	**0.010**
DCO-SB%	- 0.209 (- 0.330 – - 0.081)	**< 0.01**
DCO-VA ($mmol*min^{-1}*kPa^{-1}*L^{-1}$)	- 0.126 (- 0.250 – 0.003)	0.055
DCO-VA%	- 0.185 (- 0.305 – - 0.059)	**< 0.01**

Legend

Spearman`s correlation coefficient (*ρ*) of rank correlation (with 95% confidence intervals). The parameters of lung diffusion capacity were available in 234 patients (55%), 58 (36%) of whom had hypercapnia.

Abbreviations: ρ, Spearman`s rank correlation coefficient; FVC, forced vital capacity; FEV1, forced expiratory volume in 1 second; RV, residual volume; TLC, total lung capacity; DCO-SB, single-breath lung diffusion capacity for carbon monoxide; DCO-VA, transfer coefficient for carbon monoxide (Krogh index).

Table S4: Variables associated with chronic hypercapnia – Results of univariable and multivariable binary logistic regression analysis (all patients; N = 423)

Variables	Univariable analysis		Multivariable analysis	
	**OR (95%CI)**	***P value***	**OR (95%CI)**	***P value***
**Clinical characteristics**				
Age (years)	1.01 (0.98–1.03)	0.610	–	n.s.
Female gender	1.13 (0.75–1.68)	0.565	–	n.s.
Obesity (BMI ≥ 30 kg/m^2)	1.05 (0.57–1.90)	0.886	–	n.s.
**Pulmonary function parameters**				
FVC%	0.94 (0.92–0.95)	**< 0.01**	0.95 (0.93–0.97)	**< 0.01**
FEV1%	0.92 (0.90–0.94)	**< 0.01**	0.97 (0.94–0.99)	**0.029**
RV%	1.00 (1.00–1.01)	**0.010**	–	n.s.
TLC%	1.00 (0.99–1.01)	0.816	–	n.s.

Legend

The multivariable model used forward selection and included variables deemed clinically significant a priori (age, gender, and obesity) and those lung function parameters with a *P* value of less than 0.2 in bivariate analysis.

Abbreviations: n.s., not significant; BMI, body mass index; FVC, forced vital capacity; FEV1, forced expiratory volume in 1 second; RV, residual volume; TLC, total lung capacity.

Table S5: Variables associated with chronic hypercapnia – Multivariable binary logistic regression model (all patients; N = 423)

**Logit(p)**	L = 3.537 - 0.052 * FVC% - 0.031 * FEV1%			
**OR (95%CI); *P* value**	**FVC%**	0.95 (0.93–0.97)		< 0.01
	**FEV1%**	0.97 (0.94–0.99)		0.029
**Hosmer & Lemeshow**	0.239			
**Nagelkerke R^2**	0.287			
**AUROC (95%CI)**	0.77 (0.73 – 0.81)			
**2 x 2 table**	**TP** 92		**FP** 46	
	**FN** 70		**TN** 215	
**Metrics from 2 x 2 table (95%CI)**	**Sensitivity**	57% (48–65)	**PLR**	3.2
	**Specificity**	82% (77–87)	**NLR**	0.52
	**PPV**	67% (60–73)	**DOR**	6
	**NPV**	75% (72–79)	**F_1 score**	0.62
	**Accuracy**	73% (68–77)	**MCC**	0.40

Legend

Abbreviations: L, log-odds/Logit(p); OR, odds ratio; 95%CI, 95% confidence interval; FVC, forced vital capacity; FEV1, forced expiratory volume in 1 second; AUROC, area under the receiver operating characteristic curve; TP, true positive; FP, false positive; FN, false negative; TN, true positive; PPV, positive predictive value; NPV, negative predictive value; PLR, positive likelihood ratio; NLR, negative likelihood ratio; DOR, diagnostic odds ratio; MCC, Matthews correlations coefficient

Table S6: Linear and multiple regression models (all patients; N = 423)

**Linear regression model for FVC%**		
**Regression equation**	PCO_2 = 63.08 - 0.285 * FVC%	
**Coefficient of determination R^2**	0.219	
**F-ratio**	118.2	*P* < 0.01
**Shapiro-Wilk test**	W = 0.937	
**Linear regression model for FEV1%**		
**Regression equation**	PCO_2 = 58.06 - 0.345 * FEV1%	
**Coefficient of determination R^2**	0.172	
**F-ratio**	87.2	*P* < 0.01
**Shapiro-Wilk test**	0.917	
**Multiple regression model for FVC% and FEV1%**		
**Regression equation**	PCO_2 = 63.34 - 0.219 * FVC% - 0.120 * FEV1%	
**R^2 adjusted**	0.225	
**Variation Inflation Factor**	FVC%	2.29
	FEV1%	2.29
**F-ratio**	62.1	*P* < 0.01
**Shapiro-Wilk test**	W = 0.934	

Legend

Abbreviations: FVC, forced vital capacity; FEV1, forced expiratory volume in 1 second.
