# Supplementary material for: Hypercapnia and lung function parameters in chronic obstructive pulmonary disease
Source: BMC Pulm Med. 2024 Jul 16;24:345. doi: 10.1186/s12890-024-03151-1 (PMC11251095; doi:10.1186/s12890-024-03151-1)
Supplement: Supplementary file 2 — Supplementary Material 2. [file 12890_2024_3151_MOESM2_ESM.pdf]

Logistic regression

Dependent Y	Hypercapnia_45

Method	Forward
Enter variable if P<	0,05
Remove variable if P>	0,2

Sample size	423
Positive cases [a]	162 (38,30%)
Negative cases [b]	261 (61,70%)

[a] Hypercapnia_45 = 1
[b] Hypercapnia_45 = 0

Overall Model Fit

Null model - 2 Log Likelihood	563,016
Full model -2 Log Likelihood	462,834
Chi-squared	100,182
DF	2
Significance level	P < 0,0001
Cox & Snell R^2	0,2109
Nagelkerke R^2	0,2866

Coefficients and Standard Errors

Variable	Coefficient	Std. Error	Wald	P
FEV1_percent	-0,030845	0,014140	4,7586	0,0292
FVC_percent	-0,052048	0,010450	24,8083	<0,0001
Constant	3,53693	0,47938	54,4358	<0,0001

Variables not included in the model
age
Female_sex
BMI>30
RV_percent
TLC_percent
**DM**
**CAD**
**CHF**
**AHT**

Odds Ratios and 95% Confidence Intervals

Variable	Odds ratio	95% CI
FEV1_percent	0,9696	0,9431 to 0,9969
FVC_percent	0,9493	0,9300 to 0,9689

Hosmer & Lemeshow test

Chi-squared	10,3832
DF	8
Significance level	P = 0,2392

Contingency table for Hosmer & Lemeshow test [Hide]

Group	Y=0		Y=1		Total
	Observed	Expected	Observed	Expected	
1	41	40,067	1	1,933	42
2	36	36,938	6	5,062	42
3	30	34,035	12	7,965	42
4	33	31,203	9	10,797	42
5	35	28,730	8	14,270	43
6	24	24,798	18	17,202	42
7	19	21,740	23	20,260	42
8	15	18,524	27	23,476	42
9	17	14,516	25	27,484	42
10	11	10,449	33	33,551	44

Classification table (cut-off value p=0,5)

Actual group	Predicted group		Percent correct
	0	1	
Y = 0	215	46	82,38%
Y = 1	70	92	56,79%
Percent of cases correctly classified			72,58%

ROC curve analysis

Area under the ROC curve (AUC)	0,774
Standard Error	0,0229
95% Confidence interval	0,731 to 0,813

Tuesday, May 28, 2024 20:51

MedCalc® Statistical Software version 20.305 (MedCalc Software Ltd, Ostend, Belgium; https://www.medcalc.org; 2023)
